# Establishment and validation of an artificial intelligence-based model for real-time detection and classification of colorectal adenoma

**DOI:** 10.1038/s41598-024-61342-6

**Published:** 2024-05-10

**Authors:** Luqing Zhao, Nan Wang, Xihan Zhu, Zhenyu Wu, Aihua Shen, Lihong Zhang, Ruixin Wang, Dianpeng Wang, Shengsheng Zhang

**Affiliations:** 1 Digestive Disease Center, Beijing Hospital of Traditional Chinese Medicine, Capital Medical University, No. 23, Back Street of Art Museum, Dongcheng District, Beijing, 100010 China; 2 School of Mathematics and Statistics, Beijing Institute of Technology, No. 5, South Street, Zhongguancun, Haidian District, Beijing, 100081 China; 3 Shunyi Hospital, Beijing Traditional Chinese Medicine Hospital, Beijing, China

**Keywords:** Adaptive computer-aided diagnostic, Colorectal adenoma, Artificial intelligence, ASODE model, Real-time detection, Colonoscopy, Gastroenterology, Mathematics and computing

## Abstract

Colorectal cancer (CRC) prevention requires early detection and removal of adenomas. We aimed to develop a computational model for real-time detection and classification of colorectal adenoma. Computationally constrained background based on real-time detection, we propose an improved adaptive lightweight ensemble model for real-time detection and classification of adenomas and other polyps. Firstly, we devised an adaptive lightweight network modification and effective training strategy to diminish the computational requirements for real-time detection. Secondly, by integrating the adaptive lightweight YOLOv4 with the single shot multibox detector network, we established the adaptive small object detection ensemble (ASODE) model, which enhances the precision of detecting target polyps without significantly increasing the model's memory footprint. We conducted simulated training using clinical colonoscopy images and videos to validate the method's performance, extracting features from 1148 polyps and employing a confidence threshold of 0.5 to filter out low-confidence sample predictions. Finally, compared to state-of-the-art models, our ASODE model demonstrated superior performance. In the test set, the sensitivity of images and videos reached 87.96% and 92.31%, respectively. Additionally, the ASODE model achieved an accuracy of 92.70% for adenoma detection with a false positive rate of 8.18%. Training results indicate the effectiveness of our method in classifying small polyps. Our model exhibits remarkable performance in real-time detection of colorectal adenomas, serving as a reliable tool for assisting endoscopists.

## Introduction

Colorectal cancer (CRC) is one of the most common types of cancer and accounts for approximately 9.4% of cancer-related mortality worldwide, the incidence and mortality of CRC in the world ranked third and second, respectively, in 2020^1,2^ Adenoma is a precursor to colorectal cancer, and the development of adenoma into cancer is a common mechanism. Adenomas are frequent neoplasm found during colonoscopic screening. Detection and removal of adenomas through colonoscopy would be beneficial in preventing the development of CRCs and reducing cancer-related mortality^3,4^. The adenoma detection rate (ADR) is a well-established indicator for evaluating the capability of screening colonoscopies to detect adenomas. Theoretically, a higher ADR is more conducive to the early prevention of CRC^5^. Indeed, a 1.0% increase in ADR has been associated with a 3.0% decrease in interval CRCs^6^. However, most polyps detected during colorectal screening are diminutive in size (< 5 mm), and may therefore be easily missed by endoscopists. Recent studies have demonstrated a high degree of accuracy in detecting colorectal polyps by leveraging artificial intelligence (AI)-based models^7,8^.

Computer-aided diagnosis (CAD) technology can be separated into two categories: polyp detection and polyp classification based on the purpose of the application^9–11^. Some studies have adopted a binary classification method for polyp detection based on the presence or absence of polyps, achieving high sensitivity, which is a desired outcome^12,13^. Additionally, researchers have analyzed the statistical significance of characteristics such as the size, type, and location of polyps after detection through deep learning, further enhancing the understanding and accuracy of polyp detection methodologies^14^. For example, Rodriguez-Diaz et al. shown that the CAD model is capable of accurately distinguishing neoplastic from non-neoplastic polyps and provides a histology map of the spatial distribution of localized histologic predictions along the delineated polyp surface^15^. In addition, several studies have shown that appropriate polyp classification models can help endoscopists improve the ADR^16,17^. Ultimately, we believe that merely detecting the presence of polyps still falls short in practical applications. Appropriate classification could provide additional assistance to physicians in their assessments. Otherwise, a pathological examination is still required to determine whether the detected polyps are adenomas or other types of tumors.

Building on the success of AI-based models in achieving high accuracy for colorectal polyp detection, the next step involves refining these technologies to further enhance their diagnostic capabilities. Models such as the convolutional neural network (CNN) and You Only Look Once (YOLO) are trained on large and hard-to-obtain datasets and achieve a sensitivity above 90% and false positive rate (FPR) below 10%^18,19^. Sensitivity measures the ability of the model to correctly detect adenomas, and a good polyp classification model should have a sensitivity close to 1. The diagnosis of non-adenomatous polyps as adenomas increases the FPR. Indeed, increased sensitivity comes at the cost of increased FPR. False positive (FP) cases result in patients undergoing unnecessary surgery and are a burden on public health resources^20^. Research has achieved improvements in detection accuracy and real-time performance by integrating CSPNet, the Mish activation function, DIoU loss, and transformer blocks into the YOLOv4 architecture, and optimizing with data augmentation and ensemble learning^21^. Further research has embedded the internal structure of CSPNet into ResNet as the backbone network module for YOLOv3 and YOLOv4, providing a richer combination of gradients to enhance detection performance^22^. These modular enhancements provide a wealth of ideas for improving model quality. Additionally, incorporating the concept of ensembles into variants of the YOLO model opens up greater possibilities for enhancing model performance. For instance, integrating ResNet, GoogLeNet, and Xception into a powerful model for predicting from video frames extracted from colonoscopies significantly enhances the performance of CAD-based real-time polyp detection systems^23^. Such models trained on competition datasets or continuously acquired images have desirable classification results. However, we have shown that without specialized transfer learning, the performance of the same model on real hospital datasets may be substantially reduced. In addition, the limitations of computer performance make it impossible to train complex models.

Building on previous achievements and the potential of AI-based real-time detection and the shortcomings of existing research, this paper aims to further enhance the technical feasibility in this field. Our innovation encompasses four aspects. Firstly, we introduce an adaptive lightweight network modification and an effective training strategy that reduces the demand for computational resources in real-time detection. This approach overcomes the limitations of computer resources, allowing for predictions on low-performance devices with limited memory. Secondly, building on the lightweight network, we integrate the adaptive lightweight YOLOv4 with the SSD network to form an adaptive small object detection ensemble model (ASODE model), which improves the accuracy of target polyp detection without significantly increasing model memory. Thirdly, we extensively compare our model with the state-of-the-art YOLOv7 and YOLOv8 models, as well as the Proposed YOLOv3 and Proposed YOLOv4 models introduced in the same field, and our ASODE model outperforms these models in evaluation metrics. Finally, our model not only visualizes prediction boxes centered on polyps, classifications of polyps, and the confidence levels under predicted classifications but also runs at a speed of over 45 frames per second on ordinary devices, providing diagnostic assistance for endoscopists during real-time examinations to improve the adenoma detection rate (ADR).

## Materials and methods

### Dataset and preprocessing

Colonoscopy images were obtained from the Digestive Disease Center of Beijing Hospital of Traditional Chinese Medicine, Capital Medical University using an EVIS LUCERA CF-HQ290I endoscopy system (Olympus Optical Co, Ltd.) Polyp images were subjected to binary classification and separated into two groups containing either all clinically classified adenomas or other types of polyps, including hyperplastic and inflammatory polyp, etc. Our rationale for using binary classification was based on two factors. First, from a pathological perspective, since adenoma is a high-risk factor for CRC, our main goal was to distinguish adenomas from other types of polyps. Second, our previous model showed that multi-classification of polyps reduced sensitivity. The specific reasons and visualization results can be viewed in Supplementary Fig. S1.

The entire datasets were captured and labeled by endoscopist blinded to the subjects, which contains 1436 images and 25 videos. We divide all image datasets into training, validation, and test sets. The test set accounts for one fifth of all images, with the remaining images divided into a ratio of 9:1 for training and testing sets. Images were categorized into white light imaging (WLI) and narrow band imaging (NBI) depending on the lens type, with a resolution of 566 × 478. Videos were obtained by NBI imaging with a resolution of 720 × 576, and 50.04 frames per second. All datasets were preprocessed identically before being passed to the model. First, we scaled the images to a fixed size of 416 × 416. Then, normalization of the whole image was performed. These operations were performed automatically before the picture was passed into the model, thereby allowing application of the same model to data from different sources without further adjustment. Several raw colonoscopy images from the dataset are shown in Supplementary Fig. S2.

### Adaptive lightweight YOLOv4

The YOLOv4 model was proposed by Alexey et al. Similar to the YOLO family, the YOLOv4 algorithm innovatively treats object recognition as a regression problem, focusing on the probability of each object appearing in segmentation frames within an image. YOLOv4's output includes the probability, center coordinates, and box size for each detected object. Because the entire detection pipeline comprises a single network, it allows for the direct end-to-end optimization of detection performance. Compared to two-stage object detection algorithms, optimizing the network structure is more straightforward with YOLOv4. The design of YOLO achieves efficient object detection, offering a new paradigm for single-stage object detection methodologies. Although single-stage detection is faster, it may exhibit a slight decrease in accuracy.

The real-time application success of the YOLOv4 model has been enhanced by the improvement of several YOLOv4 algorithms that have reduced the computational cost, improved the accuracy, and reduced the inference time^24^. Compared with previous one-stage networks, YOLOv4 achieves a balance between speed and precision. However, adjusting the YOLOv4 backbone network and increasing or decreasing the number of convolutional layers only serves to improve the predictive ability of a specific dataset^25^. In addition, the inference time of the model is constrained by the number of parameters and device memory. Thus, based on the structure of the YOLOv4 network, we have proposed a strategy that reduces the number of parameters without reducing the network accuracy.

We utilized depthwise separable convolutions to reduce the number of standard convolutions. Unlike standard convolutions, depthwise separable convolutions operate in two steps. In the first step, a 3 × 3 convolution with a depth of one was applied to each channel of the input feature map. We regarded the output feature map as a whole, and the following convolutions were operated on the whole. Next, a 1 × 1 convolution was performed on the feature map with a convolution kernel equal to the number of output channels, and a convolution kernel depth equal to the input feature map^26^. The application of depthwise separable convolutions reduced the required parameters of the original convolutions by more than eight times, with only a slight decrease in accuracy. From the lightweight perspective of the model, we replaced all 3 × 3 standard convolutions in the backbone network and path aggregation network (PANet) with depthwise separable convolutions. Next, we replaced the path aggregation network (CSPNet) structure in the backbone network with an inverted residual structure. Inverted residuals divide the input feature map into two branches. One branch generates convolution layers on the input, then directly stacks it with the other branch. Inverted residuals are different from the residual network of CSPNet, and multiply the input feature map. The expansion factor determines the degree of feature map extraction. Here, we adopted the same parameter settings described in the MobileNetv2 convolutional neural network, which were our requirements for model stability. The convolution layers of inverted residuals were replaced by depthwise separable convolutions. Next, the same value as the expansion factor was used for dimensionality reduction. Finally, the manipulated branch was stacked with the branch representing the residual. The improved structure enriches the gradient information in the network output. The input and output of the inverted residual structure are referred to as bottlenecks. Since the internal convolutions are one-time tensors, then the total memory occupied by the network training is only determined by the bottleneck. We chose this structure to improve memory efficiency while slightly improving accuracy.

Restricting the upper boundary of the activation function allows the network to have good numerical resolution even when the accuracy of mobile devices is low. We replaced the unbounded Mish activation function adopted by YOLOv4 backbone with ReLU6 activation function. In addition, we removed the last activation function of the inverted residual body. Previous studies have shown that ReLU6 activation after depthwise separable convolution operation results in information loss^27^.

NetAdapt is an automated approach to network optimization that progressively reduces the resource consumption of pre-trained networks while ensuring maximum accuracy^28^. A key advantage of using NetAdapt is that it can automatically pre-train deep neural networks for mobile platforms, depending on different resource budgets. NetAdapt constructs a hierarchical lookup table and measures the resource consumption of each layer in advance to determine the number of convolutional kernels and channels reserved in a given resource-constrained layer.

Our work significantly enhances YOLOv4's efficiency for real-time detection by firstly employing depthwise separable convolutions, reducing computational load while maintaining accuracy. Secondly, we optimized the model's structure with an inverted residual mechanism, improving memory efficiency and gradient richness. Thirdly, we adopted the ReLU6 activation function for better performance on low-precision devices. Finally, we utilized NetAdapt for automated, resource-aware network optimization, ensuring optimal accuracy within resource constraints. These innovations collectively streamline YOLOv4 for practical, efficient, and accurate real-time applications.

### Single shot multibox detector (SSD)

Single shot multibox detector (SSD) is a popular and powerful target detection network^29^. We applied VGG-16 in the initial basic network to improve the performance of predicting polyps smaller than 5 mm. The SSD model encapsulates localization and detection in the forward operation of the network, thereby significantly improving the training speed of the network.

VGG-16 serves as the backbone network in the single shot multibox detector (SSD) model, playing a crucial role in feature extraction for efficient object detection. As the backbone, VGG-16 is responsible for processing input images and extracting hierarchical features of varying scales. The network's deep architecture, consisting of 13 convolutional layers with small 3 × 3 kernels, allows it to capture intricate details in the input images. In the context of SSD, these features are essential for detecting objects of different sizes. The uniformity of VGG-16's structure aids in the extraction of high-level semantic information, contributing to a more robust representation of the input scene. The features obtained from VGG-16 are then used in multiple layers of the SSD model to generate predictions for object classes and bounding box coordinates. This integration leverages the strengths of VGG-16, facilitating accurate and efficient object detection across various scales within a single pass.

### Ensemble learning

In clinical practice, most colorectal polyps are less than 5 mm. When small polyps are not detected and magnified during colonoscopy, the possibility of missed diagnosis increases. Such issues may be addressed through the development of models that improve the ability to detect small polyps, for example, by drawing prediction boxes as soon as the camera “flies by”. Ensemble learning allows both the lightweight network and small object detection ability to be taken into account^30^.

The single shot multibox detector (SSD) excels at detecting small objects, largely thanks to its data processing and transformation capabilities. Considering its rapid detection and deep feature extraction abilities, we integrated these strengths with those of the adaptive lightweight YOLOv4 to mitigate its minor shortcomings, achieving a balance between training speed and accuracy. For ease of understanding, we have named this integrated model the adaptive small object detection ensemble model (ASODE model).

The ASODE model was trained by the AdaBoost algorithm, then the model predictions were combined using the soft voting method. Soft voting enhances the accuracy and robustness of the ASODE model by assigning greater weight to more reliable models, thus leveraging the collective information from multiple models to improve overall performance and generalization capability. It ensures the integration of predictions from multiple base models based on the probability or confidence scores for each class, guaranteeing that the final prediction takes into account the confidence level of each model.

**Figure 1:**
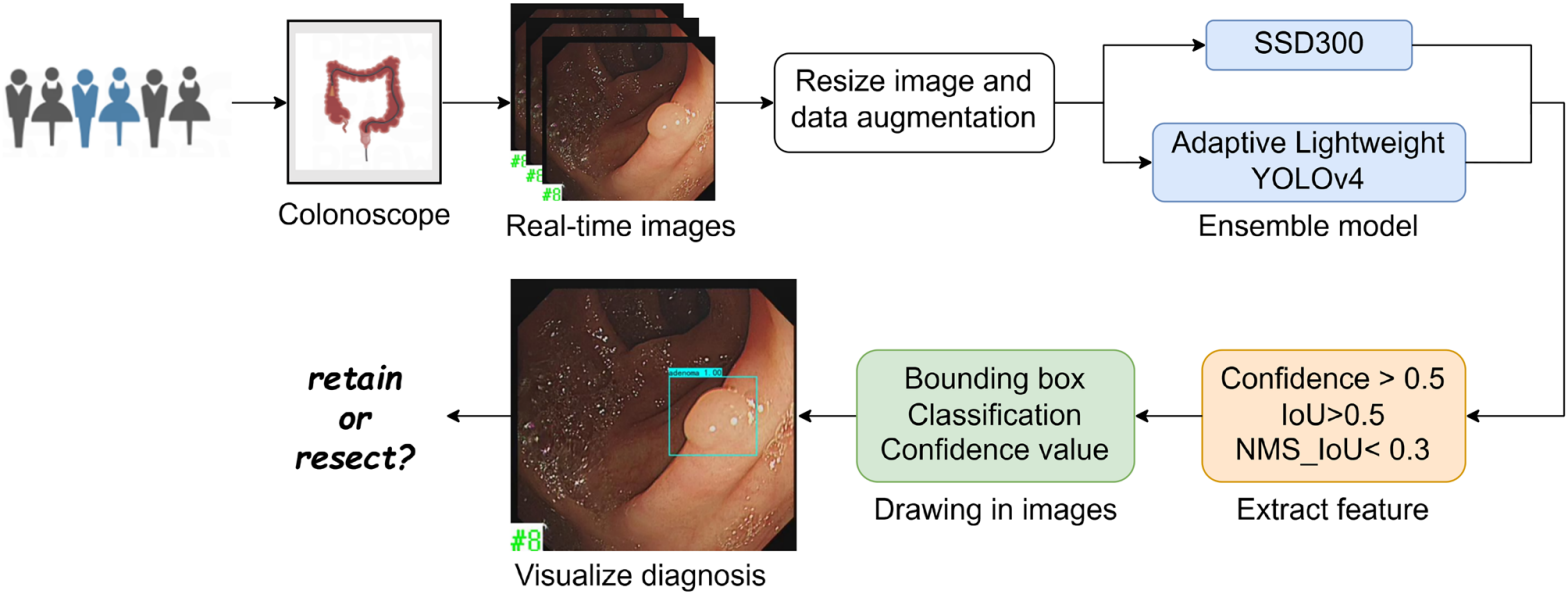
Visualization process of computer-aided clinical diagnosis.

The architectural framework of our model segment has been meticulously crafted, marking a pivotal juncture where we transition to delineate the comprehensive operational methodology of the model, as illustrated in Fig. 1.

Initially, in pursuit of constructing a robust dataset, we embarked on collecting authentic colonoscopy imagery from patients potentially afflicted with colorectal polyps. This raw data underwent a rigorous process of scaling and augmentation, ensuring a rich dataset poised for deep learning.

**Figure 2:**
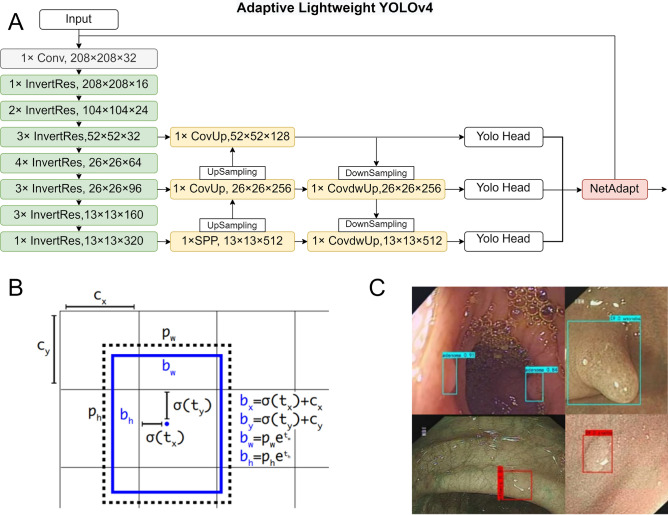
Example of the model training process. (**A**) Adaptive lightweight YOLOv4. (**B**) Parameters meaning and location of a bounding box. (**C**) Example of data augmentation.

Subsequently, these processed images are introduced into the ASODE model, a sophisticated amalgamation of adaptive lightweight YOLOv4 and SSD, designed for optimal efficiency and accuracy. The detailed structure of Adaptive lightweight YOLOv4 is shown in Fig. 2A. To refine the model's discernment capabilities further, the images undergo a deep learning training phase. This phase leverages three stringent feature extraction thresholds to ensure the preservation of only the most relevant prediction boxes, thereby filtering out any potentially misleading data.

Ultimately, the process generates a prediction box for each target, each meticulously classified and assigned a probability score. These are then seamlessly superimposed onto the original images, providing an intuitive visual representation of the model's inferences. This end-to-end methodology not only epitomizes the cutting-edge in AI-driven diagnostic tools but also significantly enhances the interpretability and applicability of our model in real-world clinical settings.

### Training techniques

In the polyp detection system, input images were divided into an $S\times S$ grid. If the center of the bounding box for a detected object fell into a grid cell, then that grid cell was responsible for calculating the classification confidence and predicting the bounding boxes. Define $p$ indicates the polyp that appears in the detection box. $Pr\left(p\right)$ represents the probability that a polyp of a specific category exists within the predicted bounding box. ${IoU}_{pred}^{truth}$ represents the Intersection over Union (IoU) between the predicted box and the ground truth box. Therefore, the $confidence(p)$ on the left side of the equation takes into account both the classification probability of polyps and the accuracy of the corresponding bounding box, and the product of the two is the confidence in predicting the existence of a certain type of polyps in the bounding box. The confidence of an object in the bounding box correspond to:

1 $$confidence\left(p\right)=Pr\left(p\right)\times {IoU}_{pred}^{truth}$$

2 $$IoU=\left|\frac{B\bigcap {B}^{gt}}{B{\bigcup B}^{gt}}\right|$$

where $B$ is the predicted box and ${B}^{gt}$ is the target box. If no polyps were present in the predicted box, then $Pr\left(p\right)$ should be zero. Otherwise, the confidence scores were equal to the intersection over union ($IoU$) between the predicted box and the target box according to (2). Bounding box predictions depended on 4 parameters, ${b}_{x}$, ${b}_{y}$, ${b}_{w}$, and ${b}_{h}$, which are defined in (3)–(6) below:

3 $${b}_{x}=\sigma \left({t}_{x}\right)+{c}_{x}$$

4 $${b}_{y}=\sigma \left({t}_{y}\right)+{c}_{y}$$

5 $${b}_{w}={p}_{w}{e}^{{t}_{w}}$$

6 $${b}_{h}={p}_{h}{e}^{{t}_{h}}$$

where ${b}_{x}$ and ${b}_{y}$ are the predicted center coordinates of the bounding box, ${b}_{w}$ and ${b}_{h}$ are the predicted width and height of the bounding box. Specifically, ${t}_{x}$ and ${t}_{y}$ are the network's output predictions for the center of the bounding box relative to the grid cell. They are constrained between 0 and 1 after being processed through the sigmoid function $\sigma \left(\cdot \right)$, ensuring that the center coordinates of the bounding box fall within the current cell. ${c}_{x}$ and ${c}_{y}$ are the top-left coordinates of the cell assigned to the current bounding box. ${t}_{w}$ and ${t}_{h}$ represents the network's output predictions for the width and height of the bounding box in log scale. ${p}_{w}$ and ${p}_{h}$ are the preset anchor dimensions for width and height, used to scale the predicted bounding box based on the network's output. Therefore, the actual width and height are calculated by exponentiating these values and multiplying by the corresponding anchor dimensions. This process adjusts the predicted width and height to match the scale of objects as they appear in the input images, allowing the model to predict bounding boxes that closely fit the objects' actual sizes. Prediction of the polyp size and location of a bounding box are shown in Fig. 2B.

A non-maximal suppression (NMS) method was used to ensure that each object was surrounded by only one prediction box. The highest classification confidence was reserved for each predicted box containing polyps. YOLO defined a confidence threshold. If the $IoU$ between two bounding boxes was greater than the threshold, then the lower confidence bounding box was eliminated. If the $IoU$ between two bounding boxes was not greater than the threshold, then both bounding boxes were reserved. Thus, no redundant bounding boxes were reserved, and the final reserved bounding boxes were the predicted boxes.

At the end of the training process, a loss function was calculated to evaluate the predicted box. Most object detection algorithms use the $IoU$ to determine the degree of overlap between the predicted box and the ground target box defined by (7). However, the bounding box regression function should consider three geometric factors: overlap area, center distance and aspect ratio. $CIoU$ loss function takes into account these factors to produce a fast convergence rate, which is formulated by (8).

7 $${LOSS}_{IoU}=1-IoU$$

8 $$LOSS_{CIoU} = 1 - IoU + \frac{{\mathop{b}\limits^{\rightharpoonup} - \overset{\lower0.5em\hbox{$\smash{\scriptscriptstyle\rightharpoonup}$}}{{b^{gt} }}^{2} }}{{c^{2} }} + \alpha v$$

where $$\mathop{b}\limits^{\rightharpoonup}$$ and $$\overset{\lower0.5em\hbox{$\smash{\scriptscriptstyle\rightharpoonup}$}}{{b^{gt} }}$$ are the center points of the predicted box $$B$$ and target box $${B}^{gt}$$, $$\rho \left(\cdot \right)$$ is the Euclidean distance and $$c$$ is the diagonal distansce between the minimum closure regions of two boxes. A positive trade-off parameter is denoted by $$\alpha$$, and $$v$$ quantifies the aspect ratio consistency according to (9) and (10).

9 $$\alpha =\frac{v}{1-IoU+v}$$

10 $$v=\frac{4}{{\pi }^{2}}{(arctan\frac{{w}^{gt}}{{h}^{gt}}-arctan\frac{w}{h})}^{2}$$

where $w$ and $h$ are the width and height of the predicted box, and ${w}^{gt}$ and ${h}^{gt}$ are the width and height of the target box.

### Evaluation metrics

The generalization performance of the model was reflected in the task requirements. We used true positive (TP), false negative (FN), FP, and true negative (TN) metrics to evaluate the model and determine whether the predicted box falls on the polyps. Consequently, we employed metrics such as precision, recall, F1 score, mean average precision (mAP), and accuracy to delineate the model's performance as outlined in Eqs. (11) through (15).

11 $$Precision=\frac{TP}{TP+FP}$$

12 $$Recall=sensitivity=\frac{TP}{TP+FN}$$

13 $$F1=\frac{2\times P\times R}{P+R}$$

We also used the mean average precision (mAP) metric, which is commonly used in target detection. The mAP averages AP values across all categories as shown in Eq. (14). Therefore, the performance of the model can be measured by a single metric

14 $$mAP=\frac{{\sum }_{q=1}^{M}AveP\left(q\right)}{Q}$$

where $Q$ is the number of polyp classifications, and $AveP\left({\text{q}}\right)$ is the average precision for a given query.

Finally, accuracy measures the proportion of correct polyp classifications over all ground truth (GT). Here, ground truth refers to total number of samples, providing a benchmark for evaluating model performance.

15 $$Accuracy=\frac{TP+TN}{GT}$$

### Data augmentation

We applied different data enhancement methods to the two sub-models in the ASODE model. Each training image is randomly sampled using a selected data augmentation method. Data augmentation simulated polyps that were not closely detected by the lens, images that were selected according to the overlap strategy, or a patch were randomly sampled from the training images. Our small dataset had a significant advantage after data enhancement, because introduction of this new “extended” data augmentation technique generated more training images.

For adaptive lightweight YOLOv4, the Mosaic data augmentation algorithm was used to enrich the detection dataset and increase the robustness ability of the model. In the training process, four images were randomly selected, flipped, scaled, and the color intensity was randomly changed. Finally, an augmented image was generated by distributing the images along the four corners. The robustness of the network was increased through the use of a random scaling operation, which added multiple small targets. In addition, the stitched images increased the batch size, and improved the efficiency of batch normalization. An example of data augmentation is shown in Fig. 2C. The images here represent the results identified after model training. The blue boxes indicate adenomas, while the red boxes denote other types of polyps.

For SSD, one of the three methods of data augmentation was selected for each training image sampled patches with a minimum overlap of 0.1, 0.3, 0.5, 0.7, or 0.9 between the sampled image and the target polyp, or randomly sampled patches. Data augmentation strategies have been shown to significantly improve performance, especially on small datasets like PASCAL VOC^30^.

### Training parameters

Training was divided into two phases: the freeze phase and the unfreeze phase. This setting reduces the memory consumption and helps to leave the local optimal solution. Adam, an adaptive learning rate optimization algorithm designed specifically for training deep neural networks, was used in our model. Unlike the stochastic gradient descent (SDG), which may be trapped in the local optimal solution during training, Adam introduces the first-order momentum and second-order momentum, respectively, such that the adaptive learning rate can improve the optimization efficiency. Although Adam can fail to converge due to too small a learning rate in later iterations, this did not occur in our study. Here, the momentum was set at 0.9, and weight decay was set at 0.0005, while other parameters were used as previously described in the YOLOv4 study^24^. In the prediction process, we retained a predicted box with classification confidence and intersection over union (IoU) greater than 0.5, while the non-maximal suppression (NMS) value was 0.3. Sample sizes were not checked using power analysis because the present study did not report statistical analysis results for between- or within-group variables. The parameters of the model are shown in Supplementary Table S1.

## Results

### Collection and examination of the image datasets

**Figure 3:**
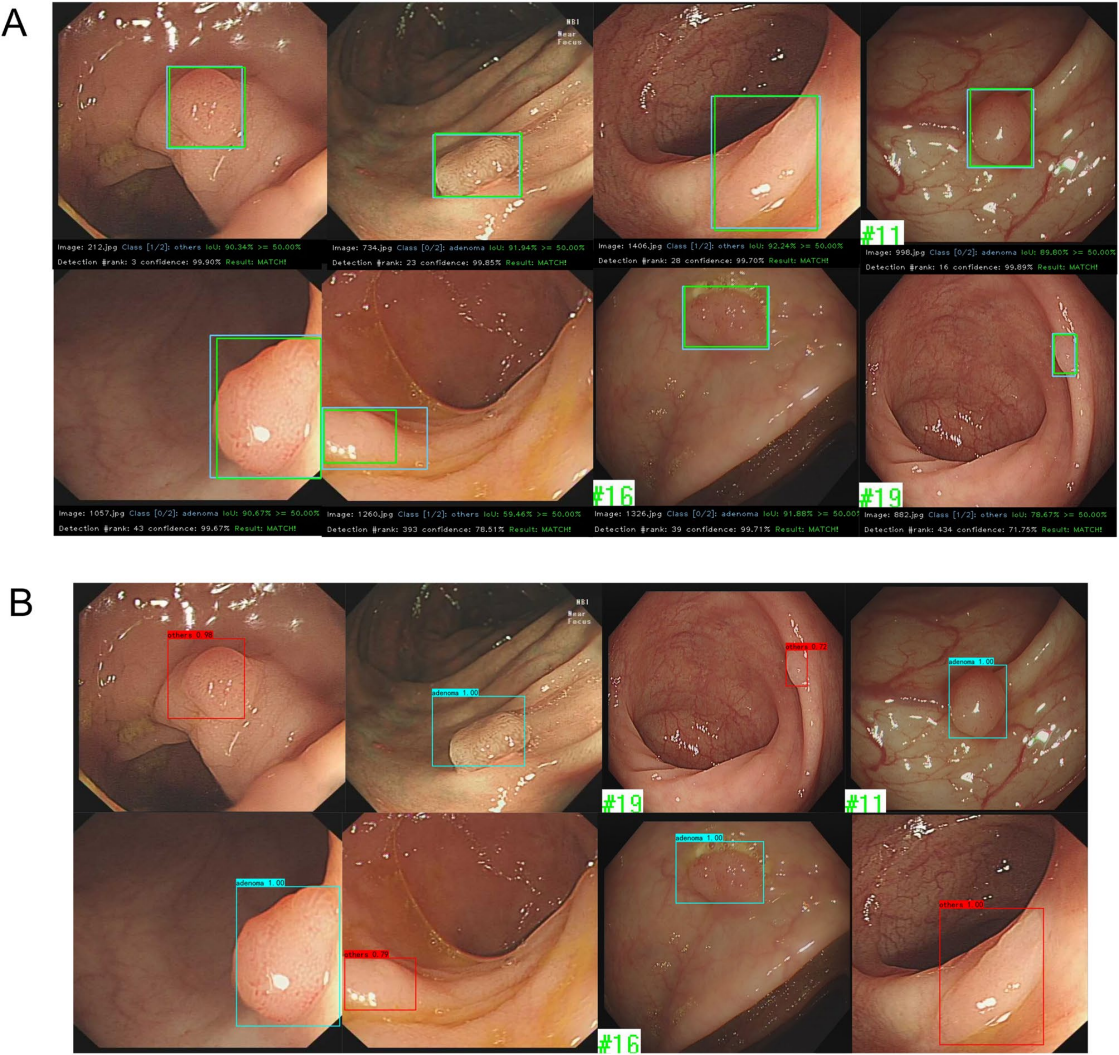
(**A**) Visualization of the conditional prediction process. The blue box represents the target box and the green box represents the predicted box. The probability of the IoU and polyp classification were calculated at the bottom of the screen. The prediction boxes that met the three threshold conditions were eventually adopted and presented in the screen. (**B**) Model prediction and associated confidence scores.

A total of 1436 polyp images were collected from 1288 patients, with 1438 videos, ranging in age from 18 to 89. 20% of the images were randomly selected for model validation. Training revealed that the following three conditions should be simultaneously met when a detection result was considered to be a TP. First, the confidence score should be greater than the confidence threshold. Second, predicted box classification should match classification of ground truth. Third, the IoU of the predicted box should be greater than the set threshold of 0.5. The three conditional prediction processes are shown in Fig. 3A.

Since our primary goal was to improve the ability to detect adenomas, additional attempts were made to reduce the FPR by training the original YOLOv4, adaptive lightweight YOLOv4, SSD and ASODE model, respectively. Detailed training and validation results including precision, recall, F1-score, and mAP are shown in Table 1. The Training is the result based on the training set and the validation set, and the Validation is the result based on the test set. In the training results, our analysis revealed improvements in the ASODE model's performance metrics compared to the lightweight YOLOv4 model: precision increased by 0.45%, sensitivity (also known as recall) by 1.2%, F1-score by 0.84%, while mean average precision (mAP) saw a decrease of 2.15%. However, accuracy experienced a notable boost of 3.22%.

**Table 1:** Training and validation results of the models.

Model	Stage	Precision%	Recall%	F1-score%	mAP%	Accuracy%
YOLOv4	Training	87.08	67.55	76.15	65.06	82.79
Adaptive light-weight YOLOv4	Training	84.79	82.61	83.68	**80.73**	81.44
SSD	Training	83.70	75.73	79.52	71.10	75.97
ASODE model	Training	**85.24**	**83.81**	**84.52**	78.58	**84.66**
YOLOv4	Validation	84.88	83.80	84.16	**86.49**	**93.91**
Adaptive light-weight YOLOv4	Validation	89.25	80.76	85.15	76.36	85.80
SSD	Validation	82.39	76.29	80.76	74.76	83.71
ASODE model	Validation	**90.29**	**87.96**	**89.38**	85.31	88.29

Significant values are in bold.

In the validation results, we observed notable improvements in the precision, sensitivity (recall), F1-score, mAP and Accuracy of the ASODE model compared to the lightweight YOLOv4 model, with increases of 1.04%, 7.2%, 4.23%, 8.95% and 2.49% respectively. We found that the ASODE model performed better than any single model in each evaluation index. Although the adaptive lightweight YOLOv4 achieved a higher mAP in the training results, this was due to the ASODE model having a larger prediction box, which reduced the IoU when the SSD classifier was used. SSD is more sensitive during the detection of small objects. However, if the prediction box is too small, the real polyps will be masked. Thus, we set a larger prediction box without changing the prediction center, so that the drawing line would not impact the endoscopists’ judgement.

**Figure 4:**
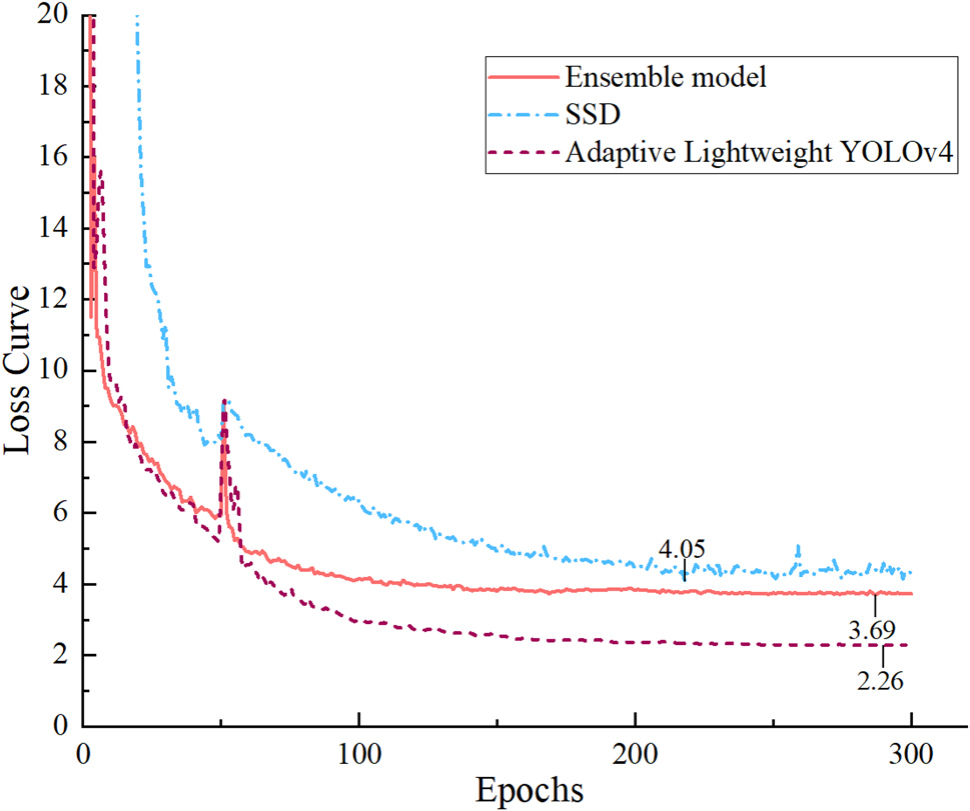
The comparison of the loss curves during the training process for the single Adaptive Lightweight YOLOv4, SSD, and the ASODE model, which highlights the strengths and weaknesses of each approach.

Furthermore, to more clearly demonstrate the training process, we will compare the validation loss curves during training using the single Adaptive Lightweight YOLOv4, SSD, and the ASODE model as shown in Fig. 4. It is evident that all three models show a significant increase in loss around the 50th epoch, followed by a rapid decrease, and eventually, all decrease to relatively low levels. Among them, the Adaptive Lightweight YOLOv4 experiences the fastest loss reduction and the lowest loss, but its loss values are the least stable; the increase in loss for the SSD is not as pronounced, and it does not converge to the relatively lowest loss; the ASODE model combines the advantages of both, with a stable loss reduction process, although the loss value obtained after 300 epochs is only intermediate between the two single models. In general, it is difficult to determine which model is more suitable for colonoscopy image detection only through training loss, so it is necessary to calculate the index and compare the training loss comprehensively.

Polyps detected during colorectal screening that are smaller in size (< 5 mm) may be missed by an inexperienced endoscopist. In our dataset, polyps smaller than 5 mm constitute over 50% of the total (regardless of adenomas or other types). The results show that most of the contribution of the model performance improvement comes from the small polyp detection ability based on the ensemble model. In the ASODE model, if the prediction box is too small, the real polyps will be masked. Thus, we set a larger prediction box without changing the prediction center, so that the drawing line would not impact the endoscopists’ judgement.

We then replicated two recently developed model, named Proposed YOLOv3 and Proposed YOLOv4, and trained it using our dataset. The training outcomes revealed that the sensitivity of the ASODE model surpassed the previously reported value of 82.80% in state-of-the-art methods by 1.48%^22^, indicating alignment with the objectives of computer-aided systems design. The mAP metric provided a comprehensive evaluation of the model's localization and classification capabilities. Similar to our training results, despite the strategy of expanding the prediction box of the ASODE model leading to a reduction in IoU, it resulted in an improvement in the model's classification ability.

Although the dataset we used was small compared to other studies, our network performance exceeded that of recent studies^22^. Firstly, we replicated the current best-performing model for training and used our dataset. Our results showed that the network structure and parameters did not achieve the expected training effect. From the perspective of network weight, the number of parameters in our ASODE model was 26,151,824 compared to 93,198,365 parameters for Proposed YOLOv3. From the perspective of evaluation index, the sensitivity of our ASODE model was 87.96% compared to 79.49%% and 82.80% for Proposed YOLOv3 and Proposed YOLOv4, respectively. The main reason for the low sensitivity of our analysis is that the model relies on large datasets for training and is not suitable for small datasets of real scenarios.

Secondly, we compared our proposed ASODE model with the newly introduced YOLOv7 and YOLOv8 methods, and the results on the test set were not ideal. Specifically, both YOLOv7 and YOLOv8 exhibited extremely high false positive rates. Although YOLOv7 and YOLOv8, like most models, have normal accuracy, meaning that most of the true classified polyps were detected, there was a very high rate of false positives, i.e., an explosion of FPR, leading to these discordant results.

Typically, the performance of the latest models would not be poor, and the cause of these results might stem from the limited number of images in our dataset. We cannot deny that YOLOv7 and YOLOv8 have shown excellent results on their released test datasets, but the applicability of the dataset still needs to be demonstrated through experiments like those presented in this paper^31,32^. Lastly, experience has shown that increasing the size of the training dataset is beneficial for improving the training effect. Thus, we anticipate that our model will be further enhanced after expanding the training dataset.

Our analysis utilized confusion matrices and False Positive Rate (FPR) data from both our models and those replicated to predict variations in polyp identification, as illustrated in Table 2. These assessments were conducted using a test set distinct from the training dataset. The ASODE model demonstrated notable accuracy rates of 92.70% for adenomas and 81.45% for other polyps, alongside an FPR of 8.18%. This indicates that our model notably lowers the FPR while maintaining high sensitivity, thereby meeting clinical objectives efficiently without the unnecessary expenditure of resources.

**Table 2:** Confusion matrices assessing the performance of the AI models, including the realistic performance of different models in the test set.

	YOLOv4		Adaptive light-weight YOLOv4		SSD	
	Predicted class		Predicted class		Predicted class	
	Adenoma	Others	Adenoma	Others	Adenoma	Others
True class						
Adenoma	145	28	165	39	151	47
Others	26	133	20	112	32	119
Accuracy (%)	92.95%	95.00%	92.36%	77.68%	88.43%	78.41%
FPR (%)	–	16.35%	–	15.15%	–	21.19%

	Ensemble model		Proposed YOLOv3		Proposed YOLOv4	
	Predicted class		Predicted class		Predicted class	
	Adenoma	Others	Adenoma	Others	Adenoma	Others
True class						
Adenoma	178	12	124	32	130	27
Others	9	101	26	138	20	102
Accuracy (%)	92.70%	81.45%	88.54%	78.23%	65.69%	53.43%
FPR (%)	–	8.18%	–	15.85%	–	16.39%

	YOLOv7			YOLOv8		
	Predicted class			Predicted class		
	Adenoma	Others		Adenoma	Others	
True class						
Adenoma	65	55		67	62	
Others	62	43		70	53	
Accuracy (%)	76.47%	68.25%		78.82%	84.12%	
FPR (%)	–	59.05%		–	56.91%	

Accuracy and FPR describe the predictions for adenomas and other types of polyps.

Moreover, the training speed of our model notably surpassed that of conventional object detection frameworks. Specifically, the ASODE model required only 2875 s to complete 300 epochs of training, in contrast to 5112 s for YOLOv4 and 5955 s for the proposed YOLOv3 to achieve the same. The output data from our model included both the prediction box and the associated confidence score, as depicted in Fig. 3B.

### Analysis of video datasets

Lens movement during clinical testing causes considerable quality bias compared to still images captured by endoscopists. Thus, the clinical application value of the model mainly depends on its performance during video detection. Here, we captured 25 real-time colonoscopy videos from 25 different patients, who were not part of the patient pool from which the image dataset was collected. These videos have a total duration of 3397 s and feature 39 genuine polyps, comprising 25 adenomas and 14 polyps of other types. Each colonoscopy examination video continuously records scenarios of polyps detected by the lens, rapid movements, and identification of non-polyp regions.

**Figure 5:**
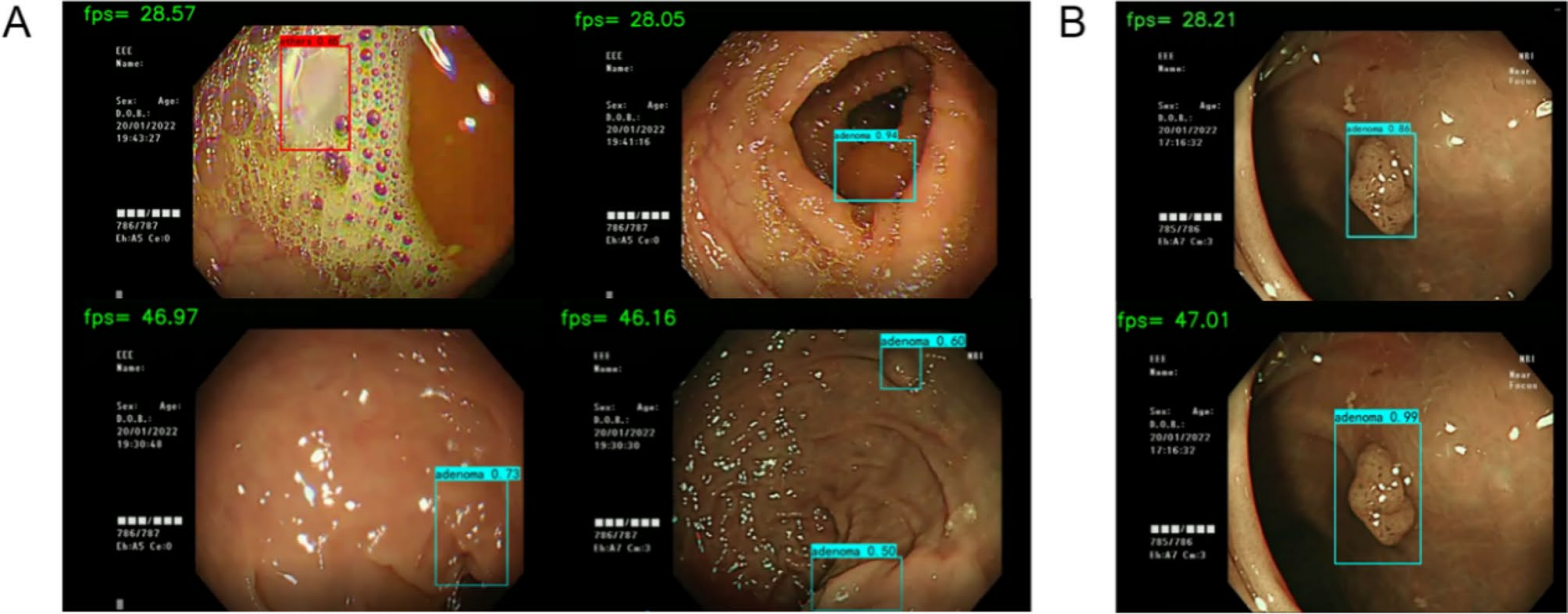
Representative data showing real-time polyp detection. Images in the first row are detected by a single SSD model, while images in the second row are detected by the ASODE model. (**A**) SSD prediction results highlight the advantages of the ASODE model after adding adaptive lightweight YOLOv4. (**B**) The ASODE model confidence is higher than that of the SSD model. A larger prediction box helps physicians to make a diagnosis macroscopically.

We conducted predictions of real-time polyp prediction using individual models and an ASODE model to compare their effectiveness. In the course of colonoscopy procedures, certain polyps may be overlooked or incorrectly identified due to a range of factors: (1) the detection system might confuse colon wrinkles and bubbles with polyps; (2) polyps that are recognized as the camera quickly passes by might suffer from variations in the confidence level of judgment and potentially in their classification due to frame changes. Illustrations of scenarios leading to potential misjudgments by the single SSD and ASODE models are depicted in Fig. 5A. Real-time predictions for all individual models can be provided upon request. Research indicates that augmenting the dataset with more negative samples could aid in diminishing the false positive rate (FPR) during prediction processes.

The ASODE model identified 42 “polyps” across 25 videos. Among these, there were 36 true polyps (23 adenomas and 13 polyps of other types) and 6 false polyps attributed to bubbles and intestinal peristalsis. This indicates that 2 adenomas and 1 other type of polyp went undetected. The primary errors of the ASODE model, when compared to the ground truth, occurred in mistaking colon peristalsis and bubbles for polyps. When the lens focuses, the model automatically corrects, not affecting the endoscopist's operational efficiency. The video test data revealed an accuracy of 92.31% and FPR score of 7.14%, which exceeded values reported in previous studies. The classification and evaluation indicators of polyps detected by video are shown in Table 3.

**Table 3:** Analysis of video datasets.

	Adenoma	Others	Total
Ground truth	25	14	39
Polyps identification by ASODE	27	15	42
Precision%	85.19%	86.67%	85.71%
Sensitivity %	92.00%	76.47%	86.42%
F-1 score%	88.46%	81.25%	86.06%
Accuracy %	92.00%	92.86%	92.31%

In addition, we found significant increases in the speed of the ASODE model in real-time prediction. The original video we used was 50.04 frames. The ASODE model could make predictions in almost every frame, with a real-time detection delay of about 20 ms, which significantly exceeded single model. A comparison between the single SSD and ASODE models in real-time prediction is shown in Fig. 5B. The increased speed of the ASODE model means that the model will not be misguided due to identification delays, and the length of the colonoscopy operation will be reduced.

## Discussion

CRC is a major public health problem due to its high mortality and morbidity. Over the past half century, adult CRC mortality and incidence have declined dramatically (51% and 32%, respectively), largely due to CRC screening and removal of adenomas^33^. With the in-depth research and breakthrough of computer vision deep learning, optical biopsy will gradually achieve accurate prediction under colonoscopy, so that targeted pathological biopsy and resection can be performed. Early diagnosis of cancer-prone lesions reduces the damage caused by multiple biopsies and medical waste.

In this study, we improved popular deep neural networks using lightweight architecture. This architecture significantly reduced the number of parameters from 46.01 to 26.15 Mb by using ASODE model hardly changing the model performance. Our model improved the confidence of polyp classification compared with state-of-the-art models by increasing the confidence threshold from 0.25 to 0.5. From the perspective of evaluation metrics, an increase in confidence threshold will decrease the evaluation of the model. Even under stricter conditions for polyp identification, our ASODE model exhibited a sensitivity of 87.96% and an FPR of 8.18% on the test set comprising 288 images. This performance surpasses the sensitivity of state-of-the-art methods trained on our dataset (79.49%)^22^. In the analysis of video datasets, the sensitivity of the ASODE model was found to be 92.31%. Our real-time polyp detection system ran with a latency of approximately 20 ms on a common personal computer (PC). The real-time detection of the model was over 47 frames. Thus, the ASODE model achieved a balance between network weight and sensitivity.

From a practical perspective, our model is not constrained by the performance of the computing platform. Even if the operational memory of the polyp detection device is not ideal, our model can work with computational efficiency in real-time detection for result display. The ultimate goal is to assist frontline healthcare professionals in determining whether polyps should be retained or excised. Lastly, our model does not require training on large-scale datasets, as readily available clinical datasets have already achieved improvements in the Adenoma Detection Rate (ADR).

There are some limitations associated with our study. For example, video datasets contain a limited number of polyps. If several hundred videos were used for testing, it might yield results more conducive to interpretation. In addition, the successful application of real-time detection systems needs to be tested on computers with different operating systems and memory resources. Finally, in both image and video detection, our model still exhibits a small amount of confusion between adenomas and other types of polyps, as well as misinterpretations of bubbles and intestinal peristalsis. To address these issues, it is imperative not only to collect more authentic colonoscopy images to augment our dataset but also to implement additional model enhancements aimed at strengthening polyp feature extraction capabilities.

In future studies, we will develop our model further by focusing on acquisition of labeled data and optimization of the model, as well as the design of clinical validation studies. We believe that optical biopsy based on AI in real time has the potential to improve the performance of endoscopists and guide clinical decisions in a more accurate, intuitive, and user-friendly manner.

## Conclusion

This study introduces a novel integrated model for real-time detection and classification of colorectal adenomas, known as the adaptive small object detection ASODE model (ASODE). The model integrates an adaptive lightweight modification of the YOLOv4 network with the single shot multibox detector (SSD) to create an efficient AI-based ensemble framework. Our approach significantly reduces the demand on computational resources while maintaining high accuracy and sensitivity in real-time polyp detection. The ASODE model demonstrated superior performance, achieving high sensitivity and accuracy in both image and video test sets, with a lower FPR, and was also successful in tests against the state-of-the-art YOLOv7 and YOLOv8 models.

Importantly, the model is designed to be practical and adaptable across various computing platforms, ensuring its effective use even on devices with less-than-ideal operational memory. This adaptability makes it a powerful tool for endoscopists, aiding in the early detection and decision-making process for the retention or excision of adenomas, which is crucial for the prevention of colorectal cancer (CRC).

Despite its encouraging results, the study acknowledges limitations, such as the need for larger-scale testing and further optimization to enhance adenoma detection accuracy. Future work will focus on expanding the model's capabilities through the acquisition of more labeled data, reduction of false positives due to bubbles and folds, and the design of clinical validation studies. The ultimate goal is to improve AI-based optical biopsy, providing endoscopists with a more accurate, intuitive, and user-friendly aid, and improving clinical outcomes in CRC prevention.

Our findings underscore the potential of AI in revolutionizing CRC screening and treatment, paving the way for more effective, efficient, and accessible diagnostic tools that can significantly alleviate the health burden of colorectal cancer.

### Supplementary Information

Supplementary Information.

## Data Availability

The raw data for this study were generated at Beijing Hospital of Traditional Chinese Medicine, Capital Medical University. Derived data supporting the findings of this study are available from the corresponding author upon request.
