# Supplementary material for: Establishment and validation of an artificial intelligence-based model for real-time detection and classification of colorectal adenoma
Source: Sci Rep. 2024 May 10;14:10750. doi: 10.1038/s41598-024-61342-6 (PMC11087479; doi:10.1038/s41598-024-61342-6)
Supplement: Supplementary file 1 — Supplementary Information. [file 41598_2024_61342_MOESM1_ESM.docx]

**Supplementary Tables**

**Supplementary Table 1.** Polyp detection model parameters.

| **Model** | **Image size** | **Training stage** | **Epoch** | **Batch size** | **Learning rate** |
| --- | --- | --- | --- | --- | --- |
| YOLOv4/ Adaptive lightweight YOLOv4 | 416 | Model freezing | 50 | 10 | 1e-4 |
|  | 416 | Model unfreezing | 100 | 16 | 1e-5 |
| SSD | 300 | Model freezing | 50 | 16 | 2e-3 |
|  | 300 | Model unfreezing | 300 | 8 | 2e-5 |

All experiments were performed using modern Titan X (Pascal) GPUs with 12 GB of RAM and processing power of 11 TFLOPS.

**Supplementary Figures**

We previously classified the colonoscopy data into adenomas, hyperplastic polyps, and other polyps, and then trained it using the same YOLOv4 model with the same parameter configuration as now. The training results show that increasing the number of classes does not help the results and may significantly reduce the mAP. The following is a visualization of the test set.


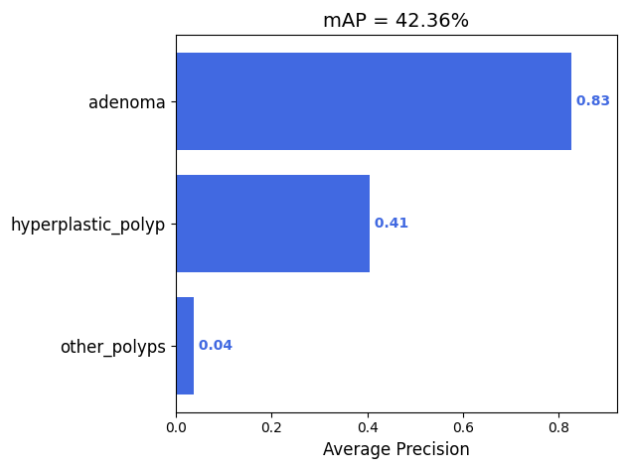

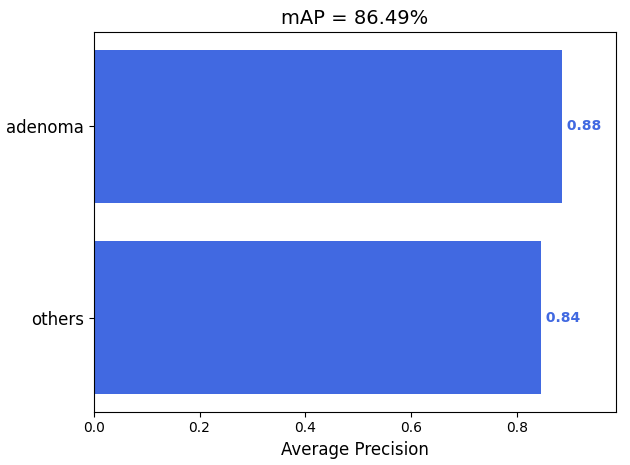


Fig. 1 mAP values and average values of each category


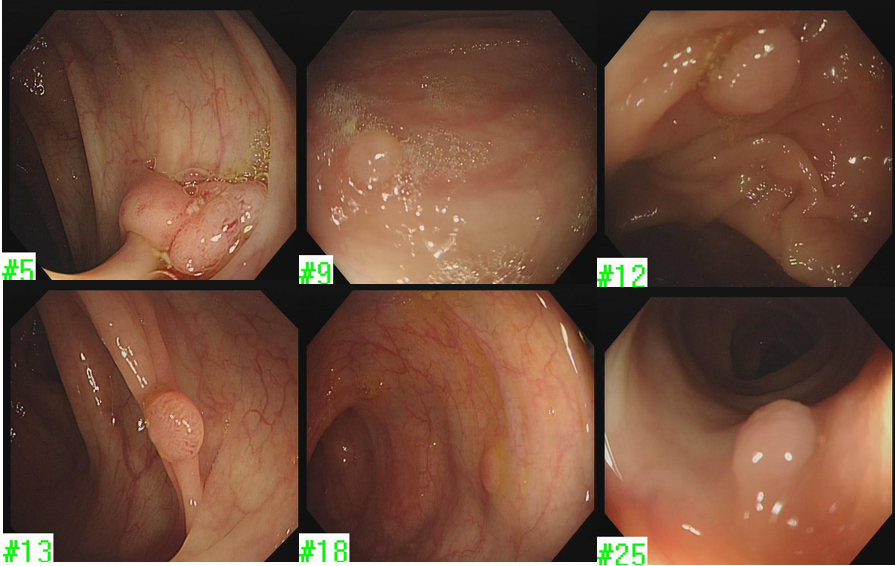


**Supplementary Fig. 2.**

Representative colonoscopy images from the dataset.
